# Family members’ conceptions of their supportive care needs across the colorectal cancer trajectory – A phenomenographic study

**DOI:** 10.1111/jan.16308

**Published:** 2024-06-28

**Authors:** Maria Samuelsson, Jenny Jakobsson, Mariette Bengtsson, Marie‐Louise Lydrup, Anne Wennick

**Affiliations:** ^1^ Department of Care Science, Faculty of Health and Society Malmö University Malmö Sweden; ^2^ Department of Pediatrics Skåne University Hospital Malmö Sweden; ^3^ Department of Surgery and Gastroenterology Skåne University Hospital Malmö Sweden

**Keywords:** colorectal cancer, conceptions, family caregivers, family members, phenomenography, qualitative, supportive care needs

## Abstract

**Aim:**

To describe the variations of family members' conceptions of their supportive care needs (SCN) across the colorectal cancer (CRC) trajectory.

**Design:**

A descriptive qualitative study with a phenomenographic approach.

**Method:**

Individual semi‐structured interviews were conducted from May 2022 to October 2022 with 23 family members of persons diagnosed with colorectal cancer. The interviews were analysed using phenomenographic analysis following the Consolidated criteria for reporting qualitative research (COREQ) checklist.

**Results:**

The phenomenographic analysis resulted in five categories. *Not of importance* describes family members' needs as unimportant due to the good prognosis and the organization of care and in relation to the needs of others. *Only satisfiable by professionals* describes information possessed by the healthcare professionals as key, as well as the need for professional counselling for the family members to process their emotions. *Managed by themselves* describes family members preferring to manage their SCN themselves by turning to the appropriate social support and/or by using coping skills. *Understood retrospectively* describes SCN as only understandable when things have calmed down and as requiring one's own experience to understand. *Left unmet* describes SCN as unnoticed by the healthcare professionals or not brought to light by the family members, or family members not knowing where to turn for support.

**Conclusion:**

Supportive care should involve individualized information, proactive and repeated assessments of needs across the trajectory, as well as encouragement of family members to reflect on their needs and to accept support when needed.

**Impact:**

There is a gap in the literature regarding family members' SCN across the CRC trajectory which this study addresses. Findings show five categories of family members' conceptions of their SCN. Those findings could serve as a basis for the development of clinical colorectal supportive care across the cancer trajectory.

**Implications for the Profession and/or Patient Care:**

Findings show that to offer family members of persons diagnosed with colorectal cancer support only at the time of diagnosis is insufficient. Instead, the healthcare team is recommended to proactively and repeatedly try to identify those in need and the characteristics of their needs. In addition, it is important to offer individualized information and strive to encourage family members to reflect on their situation and to not suppress their own needs if emerging.

**Reporting Method:**

Reporting adheres to the consolidated criteria for reporting qualitative research (COREQ) checklist.

**Patient or Public Contribution:**

No patient or public contribution.

**What does this paper contribute to the wider global clinical community?**

- Despite a worldwide growing population of family members affected by colorectal cancer, this is the first paper describing family members' conceptions of their supportive care needs across the cancer trajectory.

## 1 INTRODUCTION

Colorectal cancer (CRC) is the third most common cancer diagnosis globally, with increasing incidence and prevalence (World Health Organization, 2021). Consequently, the number of surrounding family members is also expected to rise. In addition, it is well known that the health and well‐being of family members of persons diagnosed with cancer are negatively affected (Hu et al., 2023; Ji et al., 2012; Langenberg et al., 2021). They do, for instance, have a higher risk for ischemic heart disease and stroke compared with the general population (Ji et al., 2012). Further, a recent Scandinavian study of 500,000 individuals shows that during the first year after cancer diagnosis the risk of first‐onset psychiatric disorders increases by 30% among partners of persons diagnosed with cancer (Hu et al., 2023). This calls for preventive actions. The present study therefore focuses on describing the supportive care needs (SCNs) of family members of persons diagnosed with CRC. Study findings could serve as a basis for the development of clinical CRC supportive care and support interventions targeted to timepoints along the CRC trajectory.

## 2 BACKGROUND

Family members and persons diagnosed with cancer, such as CRC, go through the cancer trajectory together (Fitch, 2008; Ohlsson‐Nevo et al., 2012). Thus, they are also affected by symptoms, side effects of treatments, and psychological changes within the person diagnosed with cancer. Since the number of persons surviving CRC is increasing, and thereby the number of family members co‐surviving CRC (World Health Organization, 2021), this study focuses on the trajectory towards survival. This trajectory is divided into the phases of diagnosis, treatment, and survival (Fitch, 2008). In the present study, the phases are outlined in accordance with the Swedish national care plan for CRC, starting with the *diagnostic phase*, referring to the time between diagnosis and surgery, followed by the *treatment phase*, referring to the time from surgery until treatment is complete, and ending with the *survival phase*. For this study, the survival phase was defined as the time initiated when the persons diagnosed with CRC and their family members were informed that treatment was successful, and no further treatment was expected. Here, *family members* refer to the definition by Wright and Leahey (2013): ‘the family is who they say they are’ (p. 55), and may thus involve biological families, next of kin, and/or friends.

Across the trajectory, family members assist the persons diagnosed with cancer in practical matters and provide emotional support (Norlyk & Martinsen, 2013; Ohlsson‐Nevo et al., 2012). Simultaneously, they strive for normality in everyday life and to meet the needs of other family members. As a result, family members report that they too need support (Norlyk & Martinsen, 2013). Research also shows that across the trajectory the family members' SCNs are not always consistent with those of the person diagnosed with CRC (Yoon & Son, 2022). Thus, it is important to identify the family members' SCNs by studying them separately. Particularly, since they continue to report unmet SCNs despite evidence of the impact of cancer on their health and well‐being (Hart et al., 2022; Wan et al., 2022; Yang et al., 2021). Consequently, the search for how to properly support family members must proceed. This is particularly important as lack of support has been suggested as contributing to the negative impact on their health (Hu et al., 2023; Ji et al., 2012) and is associated with poorer quality of life during years of survivorship (Kim et al., 2019).

This study is theoretically underpinned by the understanding of ‘SCN’ as outlined in the original Supportive Care Framework by Fitch (2008). A framework that, post the data collection of this study, was published by Krishnasamy et al. (2022) in a revised version, to refresh the concept of supportive care relevant to today's cancer care. However, on comparison the conceptual understanding of SCNs outlined in the original framework from 2008 still seems valid. That framework draws upon the Stress and coping model by Lazarus and Folkman (1984), who assert that a life event, such as a cancer diagnosis and subsequent treatment, may lead to a person's usual ways of meeting their own needs being compromised and thus not sufficient. This may require finding new ways of meeting one's needs, such as searching for information, learning new skills, or reaching out to other people. However, when the stresses of the cancer experience emerge, such new ways may be difficult to identify. As a result, some end up needing support. The Supportive Care Framework (Fitch, 2008) further explains SCNs as ranging from informational, practical, and emotional support to relational and spiritual needs. Moreover, the framework describes the SCNs as influenced by where in the cancer trajectory the family members are, and by the specific context and characteristics of the family members. Thus, a supportive care that does not recognize individual needs disclosed by family members, has been criticized for not leading to adequate actions (Fitch, 2008).

In addition, recognition of the SCNs of a specific cancer trajectory is too recommended (Samuelsson et al., 2021), which is the reason for the present study's focus on CRC specifically. The SCNs of family members of persons diagnosed with CRC are only partially described, since previous qualitative studies have focused on the experiences of being a family member of a person diagnosed with CRC (Norlyk & Martinsen, 2013; Ohlsson‐Nevo et al., 2012) rather than on explicit explorations of unmet SCNs. These studies outline the CRC trajectory as starting with a diagnostic phase, marked by shock and followed by the treatment phase, paved with caregiving, which decreases with time to return to normal after a year, yet a new normal (Ohlsson‐Nevo et al., 2012). Although, family members' quality of life is reported lowest during the first year post diagnosis (Kilic & Oz, 2019), some family members continue to have unmet SCNs even in long‐term survivorship (Kim et al., 2019).

Research describing unmet SCNs across the trajectory and involving family members of persons diagnosed with CRC, like the study by Yang et al. (2021), includes mixed cancer groups, unfortunately without the possibility to extract findings on any specific population or describes SCNs related to a certain (perioperative) event (Wan et al., 2022). Yet another study, using text mining analysis of posts written on an internet CRC self‐help support group, has identified the SCNs of patients and caregivers in each phase of their cancer trajectory (Yoon & Son, 2022). Although offering essential insights on what family members may need, none of the above studies have a phenomenographic design describing how family members think of their SCNs. Such a design offers additional insights into the understanding of family members' SCNs and how these can be addressed. Hence, there is a gap in the literature regarding family members' SCNs across the CRC trajectory that this study aims to address. Thus, the study aims to describe the variations of family members' conceptions of their SCNs across the CRC trajectory in order to inform guidance regarding the provision of tailored supportive care.

## 3 THE STUDY

### 3.1 Aim

To describe the variations of family members' conceptions of their SCN across the CRC trajectory.

## 4 METHOD

### 4.1 Design

The study is designed as a descriptive qualitative study with a phenomenographic approach. A phenomenographic study aims to map the different ways people experience a phenomenon in the surrounding world (Marton, 1986) referred to as the second‐order perspective (Marton, 1981). A second‐order approach means focusing not on the phenomenon itself (a first‐order perspective), but on people's ideas about that phenomenon – their ‘conceptions’ of the phenomenon. A conception results from a human being's thinking about their external world, and in this study, family members are thinking about their SCNs. To achieve the aim of this phenomenographic study, data were collected using individual semi‐structured interviews and analysed following the seven steps for phenomenographic analysis described by Dahlgren and Fallsberg (1991).

### 4.2 Study setting and recruitment

Recruitment was made in four outpatient surgical CRC clinics in Sweden. All sites were coherent with national standardized care plans for CRC. The primary treatment is surgery, although adjuvant treatment, for example, chemotherapy or radiation, may be needed. The care plan states that the person diagnosed with CRC should be offered structured contacts with a cancer specialist nurse at the outpatient CRC clinics at the time of diagnosis, post‐surgery, and during survivorship until five years post diagnosis, both for the planning and follow‐up of treatment and for supportive care. The SCNs of the person diagnosed with CRC are assessed verbally or using a screening tool. The care plan further states that cancer specialist nurses should offer support also for family members. However, the assignment does not state *how* to support family members, and there are no structured assessments of family members' SCNs. The contact between CRC specialist nurses and family members is reported as taking primarily place at the time of diagnosis, and later in the trajectory only on the family members' initiative and usually by telephone (Samuelsson et al., 2022).

Family members were recruited purposefully from all phases of the CRC trajectory. The purposeful sampling was undertaken using a sampling frame enabling recruitment across the trajectory. The recruitment strategy was developed together with the gatekeepers, CRC specialist nurses not involved in the study and regular meetings were held to coordinate recruitment across sites. The gatekeepers handed over a letter of information and documented what phase the person diagnosed with CRC was in. After consent from the person diagnosed with CRC, those family members accompanying them were provided with written information. The persons without accompanying family members were asked about their willingness to give the study information to a family member of their choice. Attached to the written information was a form on which family members could indicate their interest to participate and which they could then send to the first author in a pre‐paid envelope. Family members indicating an interest were subsequently contacted for verbal information and given time to ask questions. After being given time to reflect on the meaning of participation, they were asked about their willingness to participate in the study. Those giving their verbal informed consent were asked by the person responsible for the study to sign and return a written informed consent form to the research group, after which an interview was scheduled.

#### 4.2.1 Inclusion and exclusion criteria

Study participants were family members of persons diagnosed with CRC. The participants were recruited using the following inclusion criteria: family members of a person diagnosed with CRC who are >18 years old and able to read and understand Swedish. Exclusion criteria were: family members of persons diagnosed with CRC in a palliative phase, or expected to require palliative care, since research according to Christophe et al. (2022) points to SCNs being related to the expected trajectory and the present study aims to describe family members' conceptions of their SCNs across the CRC trajectory towards survival.

### 4.3 Data collection

Individual semi‐structured telephone interviews were carried out by the first author from May 2022 to October 2022. Interviews were scheduled at the participants' convenience. The family members all chose to be in their homes when interviewed. The interviewer was in a private, locked, office. The interviews lasted for as long as the participants needed and were, with the participants' permission, audiotaped and transcribed verbatim. After having conducted 20 interviews, the first author assessed the interviews for redundancy together with the last author, after which three additional interviews were performed before redundancy was confirmed and interviewing, thus, stopped.

#### 4.3.1 Interview

The interviews followed a semi‐structured interview guide (see File S1) based on previous research on family members' experiences and SCNs during the trajectory of cancer (e.g., Lavallée et al., 2019; Norlyk & Martinsen, 2013; Ohlsson‐Nevo et al., 2012; Yang et al., 2021). A pilot interview (*n* = 1) was conducted to test the interview guide and the recording equipment, leading to no changes, and the pilot interview was thus included in the dataset of the present study. The overall structure of the interview followed the CRC trajectory from the time of diagnosis to treatment and until where the family were at the time of the interview. At the start of the interview, background data were collected, which was followed by information about the study aim. Support was defined as an umbrella term comprising emotional, informational, practical, relational and spiritual support. It was, further, recognized as subjective, which is why the family members were encouraged to reflect on what it meant to them. SCN were defined in accordance with understanding of support and with Sanson‐Fisher et al. (2000), as the requirement of some action or resource that is necessary, desirable, or useful in order to attain optimal well‐being. Further, the interview sought to obtain the second‐order perspective of the family members' SCNs by asking the participants to reflect in terms of how they perceived the situation, and by using probing, prompting, and looping questions, such as ‘You said earlier that […] would you like to explain a bit further?’ or ‘Could you give an example?’

### 4.4 Data analysis

To enable a description of the variety of the family members' conceptions of their SCNs across the CRC trajectory, the transcribed data were analysed in two phases. First, an inductive phenomenographic analysis was undertaken by hand by the first and last author, guided by the seven steps described by Dahlgren and Fallsberg (1991). After this, the first author and a research librarian explored the distribution of identified categories across the CRC trajectory, using NVivo qualitative data analysis software; QSR International Pty Ltd. Version 12, 2018 (NVivo). The first author is a registered nurse from another clinical context. The last author is also a registered nurse, from an oncological context.

The phenomenographic analysis started with the transcripts being read several times regarding what they said about the family members' conceptions of their SCNs across the trajectory of CRC (Step 1: Familiarization). Then, the most significant statements were summed up (Step 2: Condensation) and contrasted (Step 3: Comparison) to identify variations and agreements. These steps were performed independently by the first and last author, respectively. Statements that seemed similar were compiled (Step 4: Grouping) through discussion between the two authors, followed by an attempt by the first author to formulate the essence of the similarity in the group of statements (Step 5: Articulation). The analysis moved iteratively between steps 3 and 5 until deemed satisfactory. The categories were thereafter named (Step 6: Labelling). Lastly, obtained categories were compared regarding similarities and differences, their relation and structure (Step 7: Contrasting). The generated categories and preliminary outcome space were then presented to the other authors, one of them having read the interviews and the other two having clinical experience from CRC care. In order to explore the distribution of identified categories across the trajectory, the transcripts were imported into NVivo, followed by the construction of a code tree based on identified conceptions and categories. Each interview (file) was coded regarding the conceptions (code) and the trajectory phases (case) and explored in a coding matrix. Findings were added to the outcome space to illustrate the distribution across the trajectory.

### 4.5 Ethical considerations

In accordance with the Declaration of Helsinki (World Medical Association, 2013), family members were informed that participation was voluntary and could be withdrawn at any time without giving any reason. In addition, written informed consent was obtained from all family members before starting the interview. To minimize intrusion, all interviews were scheduled at the participants' convenience, and in order not to cause harm the interviewer strove for sensitivity regarding the impact of the questions on the participants. The study has been approved by the Swedish Ethical Review Authority (Reg. no. 2020‐04081; 2022‐02144‐02).

### 4.6 Rigour and reflexivity

The trustworthiness criteria outlined by Lincoln and Guba (1985) were considered throughout the study design, data collection, analysis, and reporting. Credibility in a phenomenographic study concerns the relationships among the categories and the data (Stenfors‐Hayes et al., 2013), which is why quotations were integrated in the result. Further, identified categories were evaluated by a third author in relation to the transcribed interviews. Moreover, the person, with experience of qualitative interviews and the research field, conducted all interviews, and the interviews were audio recorded and transcribed verbatim, not to miss any relevant statements. The analysis was conducted iteratively and in collaboration between the authors, who all have experience of conducting qualitative analysis. Prior to data collection, the pre‐conceptions of the authors involved in the data collection and the analysis were discussed in order to minimize the influence of those pre‐conceptions on the process and description of the phenomenon. To enhance dependability, methodological choices were documented and reported using the consolidated criteria for reporting qualitative research (COREQ) checklist described by Tong et al. (2007). Further, the interview guide is accessible as a File S1. To enhance transferability, the setting and the characteristics of the participants are presented.

## 5 RESULTS

Interviews were conducted with 23 family members of persons diagnosed with CRC, 16 women and seven men, aged 29 to 85 years old. The family members were partners (*n* = 14) or adult children (*n* = 9), and from all phases of the CRC trajectory (Diagnosis *n* = 3; Treatment *n* = 6; Survival *n* = 14). More detailed characteristics and information about the treatment undergone by the persons diagnosed with CRC are found in Table 1. The interviews ranged from 25 to 64 minutes (mean 53 minutes).

**TABLE 1:** Characteristics of the participants (*n* = 23) and treatment undergone by the persons diagnosed with colorectal cancer.

Age in years, mean (range)	57 (29–85)
Gender, *n* (female/male)	16/7
Relation with the persons diagnosed with colorectal cancer, *n*	
Partner	14
Adult child	9
Level of education, *n*	
Nine‐year compulsory school	1
Upper secondary school	6
Higher education	15
Missing	1
Diagnosis	3
Treatment	6
Survival	14
Time since diagnosis in months, *n*	
0–6	9
6–12	5
12–	9
Treatment undergone by the persons diagnosed with colorectal cancer, *n*	
No surgery	3
Surgery, one	15
Surgery, multiple	5
Radiation	2
Chemotherapy	3
Stoma	4
Combination therapy	
Surgery and chemotherapy	4
Surgery and radiation	1
Surgery, chemotherapy, and radiation	1

**FIGURE 1:**
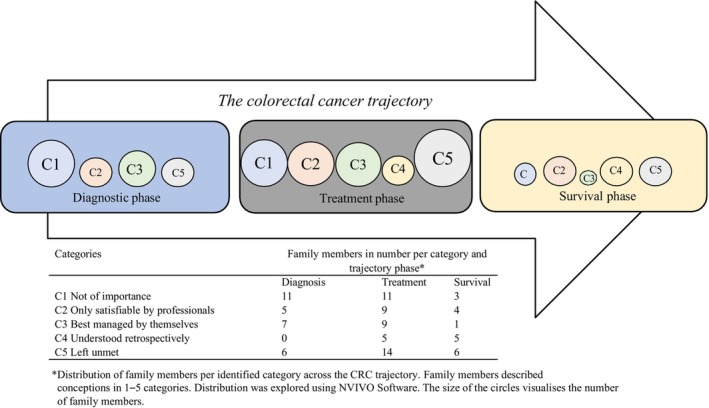
Outcome space: categories of family members' conceptions of their supportive care needs across the colorectal cancer trajectory.

The phenomenographic analysis resulted in five dominant categories of family members' conceptions of their SCNs across the CRC trajectory (Figure 1). The majority of the categories (categories 1–3, 5) were described across the trajectory, but category 4 was only described during treatment and survival. The conceptions described by family members covered one to five categories. The first category (C1), *Not of importance*, consists of conceptions of SCNs as unimportant due to the good prognosis of the illness and to the organization of care, and in relation to the needs of others. C1 was described across the trajectory although predominant during diagnosis and treatment. The second category (C2), *Only satisfiable by professionals*, consists of conceptions of the information possessed by the healthcare professionals as key to fulfilling the family members' needs and the view that processing their emotions required professional counselling. C2 was described across the trajectory. The third category (C3), *Managed by themselves*, consists of conceptions of SCNs as preferred to be handled by the family members themselves by turning to the appropriate social support and/or by using coping skills. Handling their SCNs themselves was preferred by some since they did not want to burden others with their concerns. C3 was predominantly described during the diagnostic and treatment phase. The fourth category (C4), *Understood retrospectively*, consists of conceptions of SCNs as being only understandable when things have calmed down and as requiring experience of one's own to understand. C4 was described during the treatment and survival phase. The last category (C5), *Left unmet*, consists of conceptions of SCNs as unmet due to being unnoticed by the healthcare professionals, or due to family members not bringing their needs to light or not knowing where to turn for support. C5 was described across the trajectory although predominant during the treatment phase.

### 5.1 Not of importance

In this category, SCNs were described as not being of importance due to the good prognosis of the CRC diagnosis. The good prognosis was all that mattered for the family members, which is why, during the diagnostic phase, their SCNs were described in relation to a feeling of relief. Regardless of whether the CRC diagnosis had been preceded by worries or came as a chock, in the light of expected survival their own SCNs were irrelevant. Since their needs were perceived as unimportant, during the treatment phase, they were put on hold. During this phase, it was more important to support the person diagnosed towards survival. ‘*I have to think about whether I would have said “yes, please” [to support], perhaps I would… But I didn't really feel any great need of it because for me it was more important… I mean, partly it was to see that he got going again after the operation, that it became sort of… yes. Because that was the worry then, so to speak. Yes but, what if this appetite thing lasts, if he doesn't start to eat’* (Wife, survival phase). For some family members, the conception of their SCNs as unimportant applied to the whole trajectory. For others, this changed when reaching survivorship and they understood their earlier needs (C4).

The organization of the CRC care contributed in two ways to the conception of their own SCNs as unimportant. For some family members, this was because the CRC care met all their needs. In the diagnostic phase, the combination of a clearly stated CRC care plan, a finishing line in sight (survival), and an informative and compassionate meeting with CRC healthcare professionals, was all they perceived they needed. For others, the healthcare professionals' perceived approach led them to think that there would be no need for support for family members. At the time of diagnosis, family members were informed that they did not have to take on any responsibility, they just had to come along. On the one hand, it was appreciated that the CRC care did not depend on family members' participation. On the other hand, this contrasted with their ideas of the CRC diagnosis and treatment as a family project. This, along with not being offered support, led family members to the conception that it would be an uncomplicated journey and that the healthcare professionals therefore did not expect family members to need support.

Family members' SCNs were also perceived as unimportant when contrasted with others who had it worse. Their own experiences and needs were compared with distressful stories about family members of persons diagnosed with other cancers compared to which their needs were insignificant. When contrasted with the needs of the person diagnosed with CRC, their own SCNs were even considered inappropriate. SCNs could also be ranked second to the needs of other family members. *‘But you back away a bit from receiving help yourself because you feel that you're not the one who's the worst hit, sort of’* (Daughter, treatment phase). For instance, children of a parent diagnosed with CRC could see how the other parent was suffering, and therefore their own SCNs were viewed as negligible, making them focus instead on supporting the other parent too.

### 5.2 Only satisfiable by professionals

In category 2, SCNs were described as only satisfiable by CRC healthcare professionals or professional counsellors. The family members' main focus was support for and concerns about the person diagnosed with CRC, and the information possessed by the CRC healthcare professionals was crucial. Though family members could get some information from the persons diagnosed with CRC, they were not confident about its comprehensiveness or objectiveness. They suspected that the persons diagnosed with CRC had a desire to protect them, thus choosing what to tell them. Alternatively, they assumed that the persons diagnosed with CRC did not remember all that was said. Generic CRC information found in books or on websites was also perceived as inadequate, since what they needed was information about the forthcoming trajectory of their specific family member diagnosed with CRC. Online information was even perceived as a possible source of unwelcome information that could do more harm than good. Nevertheless, family members who themselves contacted the healthcare professionals for information, performed a balancing act in order not to interfere with the privacy of the persons diagnosed with CRC. ‘*I would rather have wanted there to be somewhere I knew that I could just call and check, is this good, is it going well, is it right, sort of, does it run smoothly, will this… will it be fine? Because the alternative would have been for me to have to phone and sort of follow her disease development backwards and that would after all be quite a breach of privacy’* (Daughter, survival phase). Family members emphasized the importance of participating in meetings with the healthcare professionals, in order to get firsthand information. However, the possibility for themselves to contact the healthcare professionals was also requested.

Family members perceived supportive care from professional counsellors as superior to support from friends or other family members. ‘*And having friends, that didn't help in my case, because that was sort of about feeling sorry for me, and that's not what I wanted, you know’* (Wife, survival phase). What the family members needed was help to process what they had been through, preferably by a counsellor with knowledge about CRC. For some, the mere offer of a professional counsellor was supportive per se. Another reason for family members preferring to discuss the CRC diagnosis and treatment with professionals, rather than with other family members or friends, was to not expose the persons diagnosed with CRC – especially since aspects of symptoms of CRC and side effects of treatments were perceived as strictly private affairs. Families with minor children also desired professional support for their children, being concerned about the impact of the parents' cancer diagnosis on the children's present and future well‐being.

### 5.3 Managed by themselves

In category 3, SCNs were described as best managed by the family members themselves by turning to the appropriate social support or by using coping strategies. Hence, family members managed their SCNs by turning to the persons they preferred to be supported by. Relatives or neighbours who themselves were healthcare professionals, or themselves had experience of CRC and related treatment, were a preferred source of information. ‘*I talked to my brother, too, he is a doctor and then I had that discussion with him if something came up’* (Wife, treatment phase). However, having a physician in the family as a requirement for getting hold of desired information about the diagnosis and treatment was criticized by some for leading to unequal prerequisites for family members.

To fulfil their needs of emotional support, family members and the person diagnosed with CRC supported each other. This was described as something that could open up for deepened conversations and reinforce their relation. Alternatively, they turned to other family members, friends, or colleagues. For some, work was a source of energy where they were able to let go of their thoughts and worries, whereas for others it consumed energy they had rather spent on the persons diagnosed with CRC. However, not all had someone with whom to share emotions, or they did not want to burden others with their emotions. Hence, some strove to manage their emotional needs all by themselves by taking one day at a time, and attempting not to think and worry ahead, or by trying to find time to do things they liked, such as gardening or physical activities. It was considered important to take care of yourself in order to have strength for caregiving. Still, these activities could give rise to pangs of conscience. ‘*I have felt, you know, that they must, sort of, think that I'm stupid, working out now that… or like… here she is talking about this and then I go to the gym. But for me it's a way of coping, you know, otherwise I really wouldn't have managed this, like, if I hadn't had that refuge and sort of had an outlet for everything’* (Wife, treatment phase).

Despite healthcare professionals being considered indispensable providers of information, this was for some outweighed by an incompatible wish not to be a burden. The main priority of the family members was for the healthcare professionals to focus on the care of the person diagnosed with CRC. *‘Of course, I think it's a good thing that family members get information, but in my optimal world the focus is all on the patient, not the patient's family members’* (Son, diagnostic phase). Due to being well aware of the limited resources of the healthcare, they assumed that supporting family members too was an impossible mission. For the same reason, even family members who thought of the healthcare professionals as potential providers of support did not expect them to have the resources to proactively contact family members, even though this would have been preferred. Instead, family members contacting the healthcare professionals if needing support was considered more realistic.

### 5.4 Understood retrospectively

In category 4, SCNs were described as only understood retrospectively by the family members due to having been overwhelmed or due to the fact that understanding one's own needs required experience. Seen from the perspective of the treatment or survival phase, SCNs seemed difficult to identify and act on during the earlier phases. During the diagnostic phase, the family members had no idea of what was ahead of them and/or how they would react in the short and long term. Hence, recognition of SCNs had been impossible. Family members therefore requested extended information to all family members – information about what was expected from them post‐surgery and about the opportunity for them to take some time off from work, as well as information about practical support services.

During the survival phase, it had become evident that the diagnostic and treatment phase had been highly intense, a state of emergency, and that the family members had put their own needs aside. ‘*So, I have now, afterwards, maybe understood a little about what I actually experienced. When you, like, think like this, oh well, I'm going to be left alone. Will I be able to live here? Okay, we have cars. Yes, there are practicalities. Everything, everything. And then we had to present this to the children and talk to them. And they of course became terribly sad, terribly worried, the way you do when someone… and particularly your parent perhaps… is confronted with something? And having, sort of, the whole time had to be so… you have to be so awfully strong in order to support everyone else. Support the children, support X. You don't really have time for your own… because somewhere in all this you also have your own process that you don't really have the strength to deal with because you have to be so strong for everyone else all the time’* (Wife, survival phase). It was not till the persons diagnosed with CRC reached survival that the family members were able to focus on themselves again, recognizing both present and prior SCNs. *‘… you have re‐evaluated, you think in a different way. I mean, now I can perhaps say this, afterwards, yes, but it would have been good to have had it [support] then’* (Wife, survival phase). Family members also realized that suppressed emotional needs did not disappear by themselves, which is why they would have benefited from emotional support earlier.

Not having dealt with their SCNs earlier was perceived by the family members as making them feel worse and needing longer to heal. For some, their SCNs were not identified till seeking healthcare for physical symptoms. Still, since all their attention had been on the person diagnosed with CRC, they were not sure they would have accepted support even if previously offered. In retrospect, they understood that they had not been able to take care of themselves properly. ‘*It happened so fast. Couldn't understand then what I needed, didn't have enough energy to get in touch on my own behalf, would have needed someone to tell me* “*tomorrow at three o'clock you're going to see x”’* (Daughter, survival phase). Consequently, they wished someone else had looked after their interest for them, helped them to see their own needs, and offered support. Above all, they wished that someone had convinced them that they too were entitled to support.

### 5.5 Left unmet

In category 5, SCNs were described as left unmet, since the family members' needs were perceived as unnoticed by the healthcare professionals or/and not brough to light by themselves, or since they did not know where to turn for support. The family members' SCNs were perceived as unnoticed by the healthcare professionals as they were not assessed during the meetings, or because those needs developed when there were no contacts between family members and healthcare professionals. For instance, during surgery, they perceived the healthcare professionals to be unaware of their worries, for example, since they did not contact family members if the surgery lasted longer than expected. Further, after discharge from the hospital, the family members found themselves responsible for the recovery of the persons diagnosed with CRC, a situation they were unprepared for and therefore did not know how to deal with, yet without perceiving any support from the healthcare. *‘But you notice that now, that there are great processes around this […] you have a cancer patient, this is what should be done, this is the procedure, this is the process, these occasions, these are the numbers, contacts, and so on. So, it's worked super well. So that's what I mean, that it does make it easier. Till it just stops, after the operation, then everything just vanished. You just, but it's now that the questions come’* (Husband, treatment phase). During the survival phase, contacts with the healthcare professionals were non‐existent. Some family members were satisfied and relieved, and some had persistent SCNs. For others, their SCNs developed during this phase due to survival not turning out as expected. Fatigue, persistent stomas, and a changed relationship, for instance, were not part of the visualized survival plan drawn up during the diagnostic and treatment phase. This, along with the absence of support from the healthcare professionals, caused frustration. ‘*If you take it from a customer perspective, then we've let go of the customer here, suddenly, when their product is ready, and they've received it. No, but you've received your thing now, like that, yes, but that's not what creates value. The value is that you are done, when you are done all the way, that's what's… that's your end experience then. And here's the experience? I didn't die. That's fantastic of course, that's something to be grateful for, sort of, but…’* (Husband, survival phase). Family members requested information about how to help the person diagnosed with CRC return to a more active life, and they requested relational support, or support to process the earlier trajectory.

Family members' SCNs were left unmet because they were not brought to light by the family members themselves. Despite assurances from the healthcare of a good prognosis, for the family members the word ‘cancer’ meant death and suffering, which is why some needed to be able to prepare also for worst‐case scenarios. Yet, if the persons diagnosed with CRC did not raise such concerns, family members thought it would be improper to do so. Instead, they asked the healthcare professionals about practical arrangements, such as the length of the hospital stay during surgery, well aware that this was not what they really wanted to discuss. ‘*That I'm terrified that he will die. You know, I couldn't talk to him about it like this, well, you don't…you just don't. I'm sure we would've talked about it if we'd had another diagnosis. Then we would, like, have had to start processing and talking about what could happen and what would happen if it turned out that this couldn't… if the diagnosis had been bad. I think it would have been nice to have been offered it [support]’* (Wife, treatment phase). To enable talking about worst‐case scenarios, a private conversation with the healthcare was strongly desired across the trajectory, as well as being offered a counsellor.

Moreover, SCNs were left unmet due to family members not knowing where to turn for support. Across the trajectory, for example, after having received the diagnosis, at the time of surgery, immediately post‐surgery, or during rehabilitation or survival, family members were unsure about who to contact if needing information or clarifications.

## 6 DISCUSSION

The present study aimed to describe the variations of family members' conceptions of their SCNs across the CRC trajectory. A prevailing category found during both the diagnostic and the treatment phase was *Not of importance* (C1), which is not surprising. On the contrary, it is well known that family members place their own needs in the background, focusing on the person diagnosed with cancer (Lavallée et al., 2019; Reblin et al., 2022). In this study, however, it was revealed that the category *Not of importance* comprises three conceptions. One of those conceptions is about family members not needing support, for instance, because their needs were already met by the informational support provided by the healthcare professionals. The other two conceptions concerned family members finding their own SCNs negligible (a) because of the good prognosis of the person diagnosed with CRC and/or (b) compared with the needs of others – conceptions which meant that the family members still had SCNs. This variety of conceptions reflects the commonly described division of family members into those who do not need support and those who do (Baudry et al., 2019), where finding ways to identify the latter has been emphasized. For instance, questionnaires aiming to identify family members' unmet SCNs (Girgis et al., 2011) have been developed and validated among family members of persons diagnosed with CRC (Samuelsson et al., 2023). In addition, instruments measuring quality of life, showing promising psychometric properties (Sun et al., 2021). Further, larger cross‐sectional studies of SCNs can identify subgroups of family members. For instance, Baudry et al. (2019) have shown that by combining sociodemographic and disease‐related characteristics with outcomes of screening for distress, family members with high levels of unmet SCNs can be identified. However, when offering support for family members, the findings from the present study point to the importance of not evaluating family members' SCNs superficially or based on C1 separately, instead taking into account the multiple conceptions constituting C1, as well as the overall picture of how SCNs are perceived and related and may change across the trajectory. That is, C1 may have to do with family members not being able to identify their own needs, having no prior experience (C4). Alternatively, they may think of their needs as best managed by themselves (C3), in order not to burden anyone else. Thus, it may be beneficial if, for instance, healthcare professionals encountering family members during the diagnostic phase strive to assess their needs in a way that reveals the underlying conception of their SCNs, followed by information about the fact that this may change, and about where to turn if that happens.

A related finding of importance is that even if family members have SCNs, acting on them is not evident. One explanation from the present study was that family members were overwhelmed by the dense preparation‐program pre‐surgery and by the caregiving post‐surgery. This is in accordance with the understanding of SCNs as outlined in the Supportive Care Framework by Fitch (2008), where a stressful situation may lead to persons not having the resources required to seek help, thus needing support. The correlation between emotionally distressed family members and high levels of unmet SCNs has been previously described Baudry et al. (2019). The phenomenon that emotionally distressed and overwhelmed family members of persons diagnosed with cancer tend to underuse support services has also been previously reported (Mosher et al., 2013; Reblin et al., 2022). To explore this, Mosher et al. (2013) conducted in‐depth interviews. Their findings point to additional explanations (apart from family members being overwhelmed), for instance, negative attitudes to and inadequate knowledge of mental health services. In their study, lack of confidence in mental health professionals' ability to help led to family members not accepting an offered counsellor. Thus, applied to the CRC care context of the present study, addressing the unlikelihood of support acceptance would involve both provision of practical and informational support in order to decrease caregiver burden and, as suggested by Reblin et al. (2022), thorough and encouraging information about support services. Still, both Mosher et al. (2013) and Reblin et al. (2022) also explain family members' non‐use of support services in accordance with a conception described in C1, namely, that family members do not accept support for their own sake due to prioritizing the person diagnosed with cancer. Therefore, Reblin et al. (2022) recommend that all that intend to support family members strive to make the family members ‘shift the lens’ through which they see their own SCNs in order to make them realize that their needs too matter. One way to go could be, as suggested by one family member in the present study, the implementation of a reflective journal for family members, thereby encouraging family members to reflect on and identify their own SCNs. This could be complemented with contact information to a counsellor, either at the CRC clinic or at the healthcare center. However, in Swedish CRC care, it is not only the family members that have the person diagnosed with CRC in focus but also the CRC specialist nurses and the organization of CRC care (Samuelsson et al., 2022). Thus, it seems like a shift of lens for all involved is required, for example, by incorporating the approach outlined in the updated supportive care framework (Krishnasamy et al., 2022), where support for family members is not a detached assignment, but part of an approach that permeates the whole cancer care.

A last important finding is the conception that family members' SCNs were left unmet by the CRC healthcare professionals. Since healthcare personnel are known to be a significant source of support, both in this study (C2) and in previous studies (Norlyk & Martinsen, 2013), the CRC care may want to address this by building on the appreciated components of CRC care described in the diagnostic phase, that is, meeting with dedicated healthcare professionals, firsthand information, and a structured care plan. However, according to the findings, support for family members is needed across the trajectory. For instance, support is needed during the treatment phase, with regard to the caregiving responsibility, but also during survival, with regard to a need to process the earlier trajectory, the current situation, or the worries about recurrence. Dependent on the organization and resources of cancer care, healthcare professionals may not be in a position to support family members during this phase. Yet, it seems there is a need to inform family members that SCNs may develop during survival and about where to turn for support if that happens, in particular since SCNs were left unmet due to the family members not knowing where to turn for support. Lack of knowledge of where to turn for support during the diagnostic and treatment phase is somewhat surprising, however, as CRC specialist nurses emphasize their accessibility also for family members (Samuelsson et al., 2022), describing how they encourage family members to make contact if needing support. Hence, there is an apparent discord. A possible explanation is the shock the CRC diagnosis causes, as described by the participants in both the present and previous studies (Ohlsson‐Nevo et al., 2012). CRC healthcare professionals have previously described how they urge family members to accompany the person diagnosed to the CRC clinic, since the diagnosis is known to reduce the capacity to remember information (Samuelsson et al., 2022). However, it seemingly works the same for family members. Consequently, in trying to prevent family members' SCNs from being left unmet, those offering family members support may want to provide them with directed verbal and written information about where to turn across the trajectory, together with strategies to ensure comprehension.

### 6.1 Strengths and limitations

The study has some limitations. First, the interviews were conducted via telephone. Despite telephone interviews having been criticized for reducing the richness of qualitative data due to the absence of body language (Ward et al., 2015), studies show that telephone interviews may result in the same quality of data as face‐to‐face interviews and may be preferred by participants (ibid.). In the present study, it enabled data collection from multiple regions of Sweden as well as reducing the intrusion in the participants' everyday life.

This study had to be postponed due to the COVID‐19 pandemic, and although it was conducted post‐pandemic, there was still an impact on staffing and the burden of care of the gatekeepers. This complicated the recruitment strategy and affected the duration of the data collection. Thus, a second limitation in this study concerns the characteristics of the participants.

The original intention of the research team was to recruit a variety of family members also with regard to age and relationship to the person diagnosed but had to settle for participants from a variety of CRC clinics and trajectory phases. During recruitment, however, even these selection critierias had to be renegotiated as the gatekeepers at that time did not want to further burden upset family members. Moreover, as the persons diagnosed with CRC were invited to participate in clinical trials, inviting the family member too was experienced as too much to ask for. Thus, there is an uneven distribution of participants from the different trajectory phases – with only three persons from the diagnostic phase. To overcome this limitation, the research team proposed a prolonged recruitment period, but this was not feasible, according to the gatekeepers. However, all participants were interviewed about their experiences across the trajectory, which means that the diagnostic phase was still elucidated by all 23 family members. Retrospective reports do of course imply a risk of recall bias, and two participants exceeded 18 months since diagnosis: five and six years, respectively, although the caring responsibility was persistent. Thus, family members may have been overreporting unmet SCNs. The interviews were, however, assessed for redundancy by two members of the research team. Moreover, this study, being qualitative, did not claim to report levels of unmet needs, but to describe the variations of family members' conceptions of their SCN across the CRC trajectory.

Lastly, this study does not address the fact that vulnerable subgroups of family members may have specific SCNs as reported by Baudry et al. (2019). Given the small number of participants, we wanted to protect the confidentiality of the family members, and therefore did not collect further data on their sociodemographic or health status. Such data could, however, have increased the transferability, and their absence limits the conclusions that can be drawn. Yet, the purpose of a qualitative research study is not to generalize the findings. Nonetheless, the limitation in question points to the need for further exploring the SCNs of this population in a larger sample taking into consideration additional sociodemographic information.

## 7 CONCLUSION

How family members conceive their SCNs and prefer for them to be addressed varies. Thus, individualized support for family members is needed. Since family members regard them as key for support, healthcare professionals should be allocated resources for enabling needs assessments and adequate support. Moreover, this study suggests that only offering support at the time of diagnosis may be insufficient to support family members. Informing family members that SCNs may develop later in the cancer trajectory and where to turn if such needs occur, is essential. Especially as family members perceive their SCNs as unimportant and have no prior experience of what being a family member of a person diagnosed with CRC means. They may, further, have difficulties recognizing and acting on their needs, thus maybe declining support despite actually needing it. Family members expressing that they have no SCNs should be understood as seeing the person diagnosed with CRC as their main priority, but they may still need, or come to need, support. Therefore, healthcare professionals that encounter family members could perhaps help them realize that they too are entitled to support, as well as encouraging them to reflect on and not suppress their own needs if emerging.

## AUTHOR CONTRIBUTIONS

M.S., J.J., M.B., M.L‐L., and A.W: Made substantial contributions to conception and design, or acquisition of data, or analysis and interpretation of data; Involved in drafting the manuscript or revising it critically for important intellectual content; Given final approval of the version to be published. Each author should have participated sufficiently in the work to take public responsibility for appropriate portions of the content; Agreed to be accountable for all aspects of the work in ensuring that questions related to the accuracy or integrity of any part of the work are appropriately investigated and resolved.

## FUNDING INFORMATION

This research did not receive any specific grant from funding agencies in the public, commercial, or not‐for‐profit sectors.

## CONFLICT OF INTEREST STATEMENT

None.

### PEER REVIEW

The peer review history for this article is available at https://www.webofscience.com/api/gateway/wos/peer‐review/10.1111/jan.16308.

## Supporting information


File S1.

## Data Availability

The data that support the findings of this study are available from the corresponding author upon reasonable request.

## References

[jan16308-bib-0001] Baudry, A. S. , Vanlemmens, L. , Anota, A. , Cortot, A. , Piessen, G. , & Christophe, V. (2019). Profiles of caregivers most at risk of having unmet supportive care needs: Recommendations for healthcare professionals in oncology. European Journal of Oncology Nursing, 43, 101669. 10.1016/j.ejon.2019.09.010 31610470

[jan16308-bib-0002] Christophe, V. , Anota, A. , Vanlemmens, L. , Cortot, A. , Ceban, T. , Piessen, G. , Charton, E. , & Baudry, A. S. (2022). Unmet supportive care needs of caregivers according to medical settings of cancer patients: A cross‐sectional study. Supportive Care in Cancer, 30(11), 9411–9419. 10.1007/s00520-022-07379-7 36205779

[jan16308-bib-0003] Dahlgren, L. O. , & Fallsberg, M. (1991). Phenomenography as a qualitative approach in social pharmacy research. Journal of Social & Administrative Pharmacy, 8(4), 150–156.

[jan16308-bib-0004] Fitch, M. I. (2008). Supportive care framework. Canadian Oncology Nursing Journal, 18(1), 6–24. 10.5737/1181912x181614 18512565

[jan16308-bib-0005] Girgis, A. , Lambert, S. , & Lecathelinais, C. (2011). The supportive care needs survey for partners and caregivers of cancer survivors: Development and psychometric evaluation. Psychooncology, 20(4), 387–393. 20878835 10.1002/pon.1740

[jan16308-bib-0006] Hart, N. H. , Crawford‐Williams, F. , Crichton, M. , Yee, J. , Smith, T. J. , Koczwara, B. , Fitch, M. I. , Crawford, G. B. , Mukhopadhyay, S. , Mahony, J. , Cheah, C. , Townsend, J. , Cook, O. , Agar, M. R. , & Chan, R. J. (2022). Unmet supportive care needs of people with advanced cancer and their caregivers: A systematic scoping review. Critical Reviews in Oncology/Hematology, 176, 103728. 35662585 10.1016/j.critrevonc.2022.103728

[jan16308-bib-0007] Hu, K. , Liu, Q. , László, K. D. , Wei, D. , Yang, F. , Fall, K. , Adami, H. O. , Ye, W. , Valdimarsdóttir, U. A. , Li, J. , & Fang, F. (2023). Risk of psychiatric disorders among spouses of patients with cancer in Denmark and Sweden. JAMA Network Open, 6(1), e2249560. 10.1001/jamanetworkopen.2022.49560 36602801 PMC9857700

[jan16308-bib-0008] Ji, J. , Zöller, B. , Sundquist, K. , & Sundquist, J. (2012). Increased risks of coronary heart disease and stroke among spousal caregivers of cancer patients. Circulation, 125(14), 1742–1747. 10.1161/circulationaha.111.057018 22415143

[jan16308-bib-0009] Kilic, S. T. , & Oz, F. (2019). Family Caregivers' involvement in caring with cancer and their quality of life. Asian Pacific Journal of Cancer Prevention, 20(6), 1735–1741. 31244294 10.31557/APJCP.2019.20.6.1735 PMC7021632

[jan16308-bib-0010] Kim, Y. , Carver, C. S. , & Ting, A. (2019). Family Caregivers' unmet needs in long‐term cancer survivorship. Seminars in Oncology Nursing, 35(4), 380–383. 10.1016/j.soncn.2019.06.012 31230929 PMC6660396

[jan16308-bib-0011] Krishnasamy, M. , Hyatt, A. , Chung, H. , Gough, K. , & Fitch, M. (2022). Refocusing cancer supportive care: A framework for integrated cancer care. Support Care Cancer, 31(1), 14. 36513841 10.1007/s00520-022-07501-9 PMC9747818

[jan16308-bib-0012] Langenberg, S. , Poort, H. , Wymenga, A. N. M. , de Groot, J. W. , Muller, E. W. , van der Graaf, W. T. A. , Prins, J. B. , & van Herpen, C. M. L. (2021). Informal caregiver well‐being during and after patients' treatment with adjuvant chemotherapy for colon cancer: A prospective, exploratory study. Support Care Cancer, 29(5), 2481–2491. 10.1007/s00520-020-05738-w 32935205 PMC7981306

[jan16308-bib-0013] Lavallée, J. F. , Grogan, S. , & Austin, C. A. (2019). Cancer patients' family members' experiences of the information and support provided by healthcare professionals. Health Education Journal, 78(4), 416–427. 10.1177/0017896918812511

[jan16308-bib-0014] Lazarus, R. S. , & Folkman, S. (1984). Stress, appraisal, and coping. Springer.

[jan16308-bib-0015] Lincoln, Y. S. , & Guba, E. G. (1985). Naturalistic inquiry. Sage.

[jan16308-bib-0016] Marton, F. (1981). Phenomenography—Describing conceptions of the world around us. Instructional Science, 10(2), 177–200.

[jan16308-bib-0017] Marton, F. (1986). Phenomenography—A research approach to investigating different understandings of reality. Journal of Thought, 21(3), 28–49.

[jan16308-bib-0018] Mosher, C. E. , Champion, V. L. , Hanna, N. , Jalal, S. I. , Fakiris, A. J. , Birdas, T. J. , Okereke, I. C. , Kesler, K. A. , Einhorn, L. H. , Given, B. A. , Monahan, P. O. , & Ostroff, J. S. (2013). Support service use and interest in support services among distressed family caregivers of lung cancer patients. Psychooncology, 22(7), 1549–1556. 10.1002/pon.3168 22941782 PMC3535684

[jan16308-bib-0019] Norlyk, A. , & Martinsen, B. (2013). The extended arm of health professionals? Relatives' experiences of patient's recovery in a fast‐track programme. Journal of Advanced Nursing, 69(8), 1737–1746. 10.1111/jan.12034 23072717

[jan16308-bib-0020] Ohlsson‐Nevo, E. , Andershed, B. , Nilsson, U. , & Anderzen‐Carlsson, A. (2012). Life is back to normal and yet not – partners' and patient's experiences of life of the first year after colorectal cancer surgery. Journal of Clinical Nursing, 21(3–4), 555–563. 10.1111/j.1365-2702.2011.03830.x 21883573

[jan16308-bib-0021] Reblin, M. , Ketcher, D. , & Vadaparampil, S. T. (2022). Care for the Cancer Caregiver: A qualitative study of facilitators and barriers to caregiver integration and support. Journal of Cancer Education, 37(6), 1634–1640. 10.1007/s13187-021-02001-6 33783762 PMC8491125

[jan16308-bib-0022] Samuelsson, M. , Jakobsson, J. , Wennick, A. , Lydrup, M. L. , & Bengtsson, M. (2022). Cancer specialist nurses' experiences of supporting family members of persons diagnosed with colorectal cancer: A qualitative study. European Journal of Oncology Nursing, 61, 102205. 10.1016/j.ejon.2022.102205 36240683

[jan16308-bib-0023] Samuelsson, M. , Wennick, A. , Bengtsson, M. , Lydrup, M. L. , & Jakobsson, J. (2023). Translation, cultural adaptation, and psychometric testing of the supportive care needs survey for partners and caregivers for Swedish family members of persons diagnosed with colorectal cancer. Journal of Patient‐Reported Outcomes, 7(1), 100. 10.1186/s41687-023-00636-1 37819416 PMC10567617

[jan16308-bib-0024] Samuelsson, M. , Wennick, A. , Jakobsson, J. , & Bengtsson, M. (2021). Models of support to family members during the trajectory of cancer: A scoping review. Journal of Clinical Nursing, 30, 3072–3098. 10.1111/jocn.15832 33973285

[jan16308-bib-0025] Sanson‐Fisher, R. , Girgis, A. , Boyes, A. , Bonevski, B. , Burton, L. , & Cook, P. (2000). The unmet supportive care needs of patients with cancer. Cancer, 88, 226–237. 10.1002/(SICI)1097-0142(20000101)88:1<226::AID-CNCR30>3.0.CO;2-P 10618627

[jan16308-bib-0026] Stenfors‐Hayes, T. , Hult, H. , & Dahlgren, M. A. (2013). A phenomenographic approach to research in medical education. Medical Education, 47(3), 261–270. 10.1111/medu.12101 23398012

[jan16308-bib-0027] Sun, C. Y. , Liu, Y. , Zhou, L. R. , Wang, M. S. , Zhao, X. M. , Huang, W. D. , Liu, G. X. , & Zhang, X. (2021). Comparison of EuroQol‐5D‐3L and short form‐6D utility scores in family caregivers of colorectal cancer patients: A cross‐sectional survey in China. Frontiers in Public Health, 9, 742332. 34660519 10.3389/fpubh.2021.742332 PMC8511410

[jan16308-bib-0028] Tong, A. , Sainsbury, P. , & Craig, J. (2007). Consolidated criteria for reporting qualitative research (COREQ): A 32‐item checklist for interviews and focus groups. International Journal for Quality in Health Care, 19(6), 349–357. 10.1093/intqhc/mzm042 17872937

[jan16308-bib-0029] Wan, S. W. , Chong, C. S. , Jee, X. P. , Pikkarainen, M. , & He, H. G. (2022). Perioperative experiences and needs of patients who undergo colorectal cancer surgery and their family caregivers: A qualitative study. Support Care Cancer, 30, 5401–5410. 10.1007/s00520-022-06963-1 35298716 PMC8929239

[jan16308-bib-0030] Ward, K. , Gott, M. , & Hoare, K. (2015). Participants' views of telephone interviews within a grounded theory study. Journal of Advanced Nursing, 71(12), 2775–2785. 10.1111/jan.12748 26256835

[jan16308-bib-0031] World Health Organization . (2021). Cancer. https://www.who.int/news‐room/fact‐sheets/detail/cancer

[jan16308-bib-0032] World Medical Association . (2013). Declaration of Helsinki ‐ Ethical Principles for Medical Research Involving Human Subjects. http://www.wma.net/en/30publications/10policies/b3/index.html.pdf

[jan16308-bib-0033] Wright, L. M. , & Leahey, M. (2013). Nurses and families: A guide to family assessment and intervention. FA Davis Company.

[jan16308-bib-0034] Yang, W. F. Z. , Lee, R. Z. Y. , Kuparasundram, S. , Tan, T. , Chan, Y. H. , Griva, K. , & Mahendran, R. (2021). Cancer caregivers unmet needs and emotional states across cancer treatment phases. PLoS One, 16(8), e0255901. 10.1371/journal.pone.0255901 34379667 PMC8357113

[jan16308-bib-0035] Yoon, J. , & Son, H. (2022). Need differences by treatment phases between patients with colorectal cancer and their caregivers: A text mining analysis. Asia‐Pacific Journal of Oncology Nursing, 9(5), 100061. 35619655 10.1016/j.apjon.2022.03.013 PMC9126798

