# Supplementary material for: Family members’ conceptions of their supportive care needs across the colorectal cancer trajectory – A phenomenographic study
Source: J Adv Nurs. 2024 Jun 28;81(2):1069–81. doi: 10.1111/jan.16308 (PMC11729579; doi:10.1111/jan.16308)
Supplement: Supplementary file 1 — File S1. [file JAN-81-1069-s001.docx]

**Supplementary information. Interview guide**

*The purpose of the interview is to describe family members’ conceptions of their supportive care needs in connection with colorectal cancer*

**Background questions:** Demographic questions. **Introductory question** to all informants: *What does the word ’support’ mean to you?*

*Please tell me about what your life has been like during this period (since diagnosis, post-surgery, during rehabilitation).*

The interview subsequently aims to touch on the following themes:

| **T**  **H E M**  **E**  **S** | **Support** (Emotional and spiritual support, information and practicalities – regarding the care and privately)   - Existing - Desired - Changed since earlier phases - The coming phases / the future | **Needs** (Emotional and spiritual needs, information and practicalities – regarding the care and privately)   - Personal - Those of other family members - Changed since earlier phases - The coming phases / the future |
| --- | --- | --- |
|  | **Resources**   - Personal - Changed since earlier phases - The coming phases / the future | **Obstacles**   - Personal - External - Changed since earlier phases - The coming phases / the future |

**Concluding questions** to all informants: If you were to guide a family member *who is about to receive a diagnosis*/*whose family member is going to have an operation*/*who* *is recovering post-surgery*/*who* *is in rehabilitation*, what would you have wanted to communicate to this person “What would you have liked support for family members to look like?

**Examples of follow-up questions that will be used during the interview:**

Would you like to tell me more about…? Could you give an example of…? What did you mean before, when you said…? What does that mean to you?
